# New York Academy of Medicine

**Published:** 1887-04

**Authors:** 


### NEW YORK ACADEMY OF MEDICINE.

Regular meeting, December 16, 1886—Abraham Jacobi, M. D., president, in the chair.

*Fermentation, Putrefaction, and Suppuration.*

Dr. Herman Knapp read the paper, which he had been led to prepare from the fact that there was a very wide difference of opinion among physicians as to the importance of bacteriology—some claiming that the great majority of all diseases were of bacterial origin, while others claimed the contrary, and that bacteriology was of interest only from a scientific point of view. Dr. Knapp quoted the statistics of mortality of several of the cities of the United States for a recent week, as reported in the Boston Medical and Surgical Journal, in which it was stated that the percentage of deaths from infectious diseases was 33 ; yet this did not include the deaths from consumption, diphtheria, croup, diarrhœal affections, pneumonia, peritonitis, endocarditis, malaria, gonorrhœa, typhoid fever, skin diseases, and surgical affections. After a fair calculation, Dr. Knapp thought we were justified in drawing the conclusion, which at first surprised him, that of the total number of deaths 90 per cent. were from diseases of bacterial origin. The greatest practical advance which had been made in my department of medicine of recent years was to be seen in surgery, due to the introduction of antiseptic methods, or the prevention of suppuration, which he proceeded to show took place only through the agency of germs of bacteria.

Dr. Knapp presented some flasks in which he had repeated the experiments of Schultze, Schwam, and others, demonstrating the manner in which fermentation takes place. If germs were rigidly excluded from a fermentescible substance, fermentation would not occur ; fermentation was the decomposition of carbo-hydrates by the action of the yeast plant. He further showed that putrefaction is the splitting up of nitrogenous substances into simpler elements through the agency of germs, the germs, however, differing from those at work in the process of fermentation.

The author then proceeded to show that suppuration is also dependent upon the agency of bacteria, the substance acted upon being living nitrogenous matter, while in putrefaction the substance acted upon is dead nitrogenous matter. He asked : Does mere traumatism produce suppuration ? Do foreign bodies produce suppuration ? Do chemical agents of any nature produce suppuration ? To each of these questions he gave a negative reply, based upon experimentation with rabbits. That traumatism would not produce suppuration was proven by operations upon the eyes of rabbits—upon one eye with a perfectly clean instrument, suppuration following in no case ; upon the other eye, with an infected instrument, suppuration followed in every instance. Simple fracture would never be attended by suppuration unless there was a suppurating point at some other part of the body, the germs being transported through the circulating medium.

To prove that foreign bodies would not cause suppuration, he had left the end of a rusty hair-pin brought to a glow (thus killing all germs) in the eye of a rabbit, and suppuration had not followed; the end of the same hair-pin not brought to a glow produced suppuration.

It was generally claimed, and by the highest authority, that chemical substances will produce suppuration. Dr. Knapp thought this was an erroneous belief due to the great difficulty of experimenting antiseptically with these agents. This was especially true of croton oil, which was extremely irritating in the smallest quantity. Yet he had been able to inject a drop of this substance into the eye of rabbits and not produce suppuration. Signs of irritation within the eye followed, but not suppuration, nor were germs present. In the other eye, however, precautions against the entrance of germs with the oil not being taken, suppuration ensued. The animals were shown.

Dr. Charles Heitzman made some remarks, and said that he had examined the eye of the rabbit into which croton oil had been injected with precautions against the entrance of germs, and while he saw signs of inflammation, he could find neither pus nor germs. Yet it seemed difficult to explain the development in a person otherwise healthy of, for instance, suppurative osteo-myelitis.—*Medical and Surgical Reporter.*

